# Novel Knowledge-Based Transcriptomic Profiling of Lipid Lysophosphatidylinositol-Induced Endothelial Cell Activation

**DOI:** 10.3389/fcvm.2021.773473

**Published:** 2021-11-29

**Authors:** Keman Xu, Ying Shao, Fatma Saaoud, Aria Gillespie, Charles Drummer, Lu Liu, Yifan Lu, Yu Sun, Hang Xi, Çagla Tükel, Domenico Pratico, Xuebin Qin, Jianxin Sun, Eric T. Choi, Xiaohua Jiang, Hong Wang, Xiaofeng Yang

**Affiliations:** ^1^Centers of Cardiovascular Research, Inflammation and Lung Research, Philadelphia, PA, United States; ^2^Neural Sciences, Temple University Lewis Katz School of Medicine, Philadelphia, PA, United States; ^3^Departments of Cardiovascular Sciences, Metabolic Disease Research, Thrombosis Research, Philadelphia, PA, United States; ^4^Center for Microbiology & Immunology, Temple University Lewis Katz School of Medicine, Philadelphia, PA, United States; ^5^Alzheimer's Center, Temple University Lewis Katz School of Medicine, Philadelphia, PA, United States; ^6^National Primate Research Center, Tulane University, Covington, LA, United States; ^7^Department of Medicine, Center for Translational Medicine, Thomas Jefferson University, Philadelphia, PA, United States; ^8^Surgery (Division of Vascular and Endovascular Surgery), Temple University Lewis Katz School of Medicine, Philadelphia, PA, United States

**Keywords:** transcriptomic analysis, inflammation, secretomes, RNA-Seq analysis, aortic endothelial cell

## Abstract

To determine whether pro-inflammatory lipid lysophosphatidylinositols (LPIs) upregulate the expressions of membrane proteins for adhesion/signaling and secretory proteins in human aortic endothelial cell (HAEC) activation, we developed an EC biology knowledge-based transcriptomic formula to profile RNA-Seq data panoramically. We made the following primary findings: first, G protein-coupled receptor 55 (GPR55), the LPI receptor, is expressed in the endothelium of both human and mouse aortas, and is significantly upregulated in hyperlipidemia; second, LPIs upregulate 43 clusters of differentiation (CD) in HAECs, promoting EC activation, innate immune trans-differentiation, and immune/inflammatory responses; 72.1% of LPI-upregulated CDs are not induced in influenza virus-, MERS-CoV virus- and herpes virus-infected human endothelial cells, which hinted the specificity of LPIs in HAEC activation; third, LPIs upregulate six types of 640 secretomic genes (SGs), namely, 216 canonical SGs, 60 caspase-1-gasdermin D (GSDMD) SGs, 117 caspase-4/11-GSDMD SGs, 40 exosome SGs, 179 Human Protein Atlas (HPA)-cytokines, and 28 HPA-chemokines, which make HAECs a large secretory organ for inflammation/immune responses and other functions; fourth, LPIs activate transcriptomic remodeling by upregulating 172 transcription factors (TFs), namely, pro-inflammatory factors NR4A3, FOS, KLF3, and HIF1A; fifth, LPIs upregulate 152 nuclear DNA-encoded mitochondrial (mitoCarta) genes, which alter mitochondrial mechanisms and functions, such as mitochondrial organization, respiration, translation, and transport; sixth, LPIs activate reactive oxygen species (ROS) mechanism by upregulating 18 ROS regulators; finally, utilizing the Cytoscape software, we found that three mechanisms, namely, LPI-upregulated TFs, mitoCarta genes, and ROS regulators, are integrated to promote HAEC activation. Our results provide novel insights into aortic EC activation, formulate an EC biology knowledge-based transcriptomic profile strategy, and identify new targets for the development of therapeutics for cardiovascular diseases, inflammatory conditions, immune diseases, organ transplantation, aging, and cancers.

## Introduction

Atherosclerosis is a pathological process underlying the development of myocardial infarction, stroke, and peripheral arterial disease, which is a substantial cause of morbidity and mortality (1). Vascular inflammation contributes significantly to the atherosclerotic onset and the development of its complications (2–5). In addition to consistent findings across multiple mouse models (6), the Canakinumab Anti-inflammatory Thrombosis Outcomes Study (CANTOS) demonstrated that the inhibition of pro-inflammatory interleukin-1β (IL-1β) reduces the atherosclerotic burden in cardiovascular disease (7–9). The activation of endothelial cells (ECs) is the earliest event and a central pathological process associated with the onset of atherosclerosis. Based on our previous findings, we propose that:(1) ECs are innate immune cells (3–5), as they display innate immune functions similar to those of prototypical innate immune cells, such as macrophages (5, 10, 11) and monocytes (12–18). (2) In addition to increased secretion of cytokines and chemokines and upregulation of adhesion molecules, activated ECs also exhibit two new hallmarks of innate immune cells, namely, upregulation of both danger-associated molecular patterns (DAMPs) receptors and major histocompatibility complex (MHC) molecules for antigen presentation (19). (3) Endogenous metabolites that bind to their intrinsic receptors, rather than classical DAMP receptors, such as toll-like receptors (TLRs) and nod-like receptors/inflammasomes, can become conditional DAMPs, for example, lysophospholipids (19–23). (4) Similar to macrophages and monocytes, ECs have innate immune memory functions (trained immunity) (2, 3, 24–26). Although many transcriptomic data have been reported, there is no standard universal framework to analyze these data. To address this knowledge gap, we applied the ontology transcriptomic formula to characterize aortic endothelial cell activation.

There are four key features for conditional DAMPs as we proposed previously: (i) acting as endogenous metabolites; (ii) elevating in pathologically conditions; (iii) contributing to physiological signaling roles; (iv) binding to their intrinsic receptors and carrying out cytokine-like signal amplification functions (27). Conditional DAMPs include lysophospholipids, hyperhomocysteinemia (14–17, 28–30), and succinate (27) among others. Lysophospholipids are a group of bioactive lipids; some of which are pro-inflammatory molecules (31, 32), such as lysophosphatidylcholines (LPCs, lysoPC) (23, 33, 34), lysophosphatidic acid (LPA) (34–36), lysophosphatidylinositols (LPIs, lysoPIs) (19), and sphingosine-1-phosphate (37). LPA, LPCs, and LPIs are the characteristics of atherosclerotic aorta plaque in apolipoprotein E deficient (ApoE^−/−^) and low-density lipoprotein receptor (LDLR^−/−^) mice. One of the sub-species of LPIs, 18:0, has been reported and is mainly localized in the necrotic core of the plaque (38). In addition to pro-inflammatory molecules, we also proposed anti-inflammatory endogenous metabolites, such as lysophosphatidylethanolamine and lysophosphatidylserine, pro-resolving mediators (39), IL-35 (40), and itaconate (41, 42) as homeostasis-associated molecular patterns (20). As we have reported, most lysophospholipids (LPLs) contribute to aortic endothelial cell (EC) activation (23, 43, 44) and the progression of atherosclerosis (22). The molecular mechanisms underlying LPC-induced aortic EC activation included calcium influx-increased proton leaks *via* uncoupled mitochondrial electron transport chain, increased mitochondrial reactive oxygen species (mtROS), increased histone 3 lysine 14 acetylation (H3K14ac), and transcription factor AP-1 driven ICAM-1 upregulation (23, 43–46). In addition, we also reported that LPC induces caspase-1 activation and pyroptosis (inflammatory cell death) (33, 34, 47). Moreover, by RNA-Sequencing (RNA-Seq), we reported that LPC and LPIs induce prolonged EC activation by upregulating adhesion molecule ICAM-1, additional DAMP receptors such as CD36, and MHC molecules for antigen presentation (19). However, the transcriptomic formula of aortic EC activation in a panoramic view remained poorly characterized.

**Figure 1:**
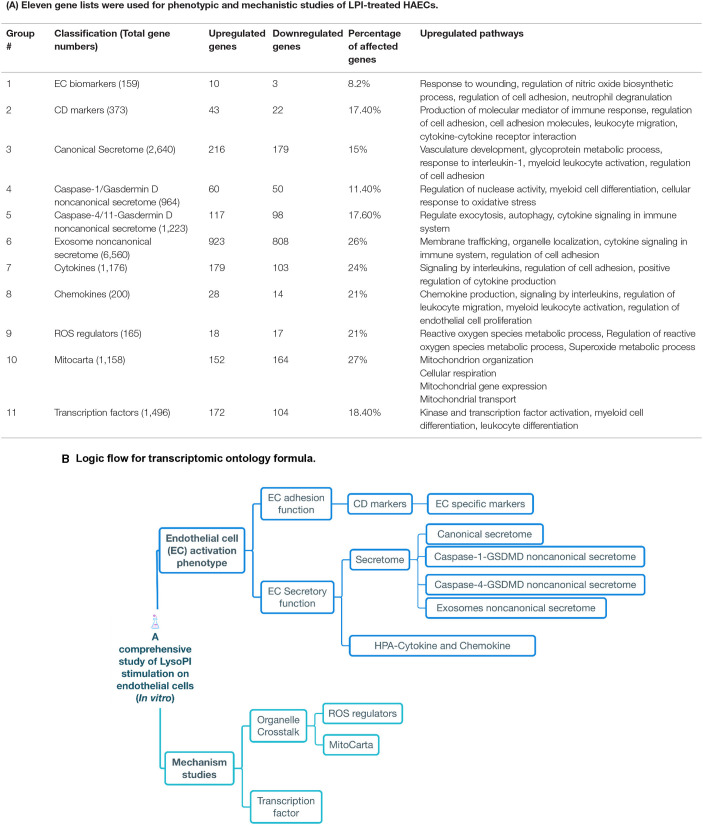
Comprehensive analysis of lysophosphatidylinositol LPI)-treated human aorta endothelial cells (HAECs) at messenger RNA (mRNA) level from 11 different aspects. **(A)** Phenotypic and mechanistic studies. The table shows how a gene changes 18 h after HAECs were treated with 10 μM LPIs. The last column indicates that the pathways are promoted by LPI-upregulated genes. Pathways associated with endothelial cell activation are marked in red. **(B)** Logic flow for our knowledge-based transcriptomic profile strategy.

Low-throughput techniques used in current cardiovascular science research laboratories limit our understanding of aortic EC activation. Thus, high-throughput computational bioinformatics screening is often introduced to provide a whole picture at the beginning of an experimental project. As an initial step, RNA-Seq data can be profiled *via* various databases, for example, Gene Set Enrichment Analysis (GSEA) (19). To improve our panoramic understanding of the importance of aortic EC activation induced by conditional DAMP proinflammatory lipid LPIs, we hypothesized that transcriptomic profiling using high-throughput RNA-sequencing data can be formulated on an EC biology knowledge basis. We examined this new hypothesis by massive profiling. Aortic EC phenotypic research was studied from EC adhesion and secretory functions. LPIs induce aortic EC activation by upregulating EC biomarkers and membrane adhesion molecules (159 genes), clusters of differentiation (CDs) signaling (373 genes), six types of secretomic gene sets, namely, canonical secretome (2,640 genes with signal peptide) (13), caspase-1-gasdermin D (GSDMD) non-canonical secretome (964 genes), caspase-4-GSDMD non-canonical secretome (1,223 genes), exosome non-canonical secretome (6,560 genes) (48), Human Protein Atlas (HPA) database-classified cytokines (1,176 genes), and HPA-classified chemokines (200 genes) (49). Three mechanistic studies were included in this article to identify molecular mechanisms underlying the upregulation of these key features of EC activation, such as increased endothelial cell membrane adhesion functions and secretory functions. We focused on determining the expression changes in a complete list of 165 reactive oxygen species regulators (ROS regulatome) (50) and 1,158 nuclear DNA-encoded mitochondrial genes (mitoCarta genes), and a complete list of 1,496 human genome-encoded TFs (49) (Figure 1), as have we reported for CD4^+^Foxp3^+^ regulatory T (Treg) cells (49). Our results have provided novel insights into aortic endothelial cell (EC) activation, formulated an EC biology knowledge-based transcriptomic profile strategy, and identify new targets for the future development of therapeutics for cardiovascular diseases, inflammations, immune diseases, transplantation, aging, and cancers (51).

## Materials and Methods

### Gene List Generation

Eleven gene lists were generated in this manuscript for phenotypic and mechanistic studies of LPI-treated HAECs (Figure 1). One hundred fifty-nine EC biomarkers were modified from PMID: 29333215; 373 CD markers, 1,176 cytokines, 200 chemokines, and 1,496 TFs were generated from PMID: 33613572; 2,640 canonical secretomes were downloaded from the comprehensive protein database Human Protein Atlas (https://www.proteinatlas.org/); 964 non-canonical caspase-1-gasdermin D (GSDMD) secretomes were generated from PMID: 18329368;1,223 non-canonical caspase-4 (humans)/11 (mice) secretomes were extracted from PMID: 28196878; 6,560 exosome secretome downloaded from a comprehensive exosome database (http://www.exocarta.org/); 165 ROS regulators were downloaded from PMID: 33154757; 1,158 human nuclear genome DNA-encoded mitochondrion genes were downloaded from the Broad Institute at MIT (mitoCarta, https://www.broadinstitute.org/mitocarta/mitocarta30-inventory-mammalian-mitochondrial-proteins-and-pathways).

### Microarray Datasets

Microarray datasets were collected from the National Institutes of Health (NIH)-National Center for Biotechnology Information (NCBI)-Gene Expression Omnibus (GEO) (https://www.ncbi.nlm.nih.gov/gds/) and ArrayExpress (https://www.ebi.ac.uk/arrayexpress/) databases and analyzed with online software GEO2R (https://www.ncbi.nlm.nih.gov/geo/geo2r/), as we have reported (3, 10, 52–54). Three GEO datasets were used in this manuscript, namely, GSE59226 (influenza virus infection), GSE 79218 (MERS-CoV infection for 0, 12, 24, 36, and 48 h), and GSE 1377 (Kaposi's sarcoma-associated herpes virus).

### Metascape Analysis

Metascape (https://metascape.org/gp/index.html#/main/step1) was used for enrichment analysis. This website contains the core of most existing gene annotation portals. Our 11 gene lists mentioned in Figure 1A were compared with thousands of gene sets and ontology databases (KEGG, MSigDB, and GO) that were defined by their involvement in specific biological processes, pathway membership, enzymatic function, and protein localization. More details about Metascape can be found in cited references (55).

### Cytoscape Analysis

The ClueGo v2.5.8 in Cytoscape (https://cytoscape.org/) v3.8.2 was applied to identify gene connections and interactions between functional terms/pathways, as we have reported (56). Eight ClueGO databases were used for our network analysis, namely, GO-Biological Process (17,776 terms/pathways with 18,058 available unique genes), GO-Cellular Component (1,975 terms/pathways with 18,983 available unique genes), GO-Immune System Process (1,195 terms/pathways with 3,625 available unique genes), GO-Molecular Function (5,468 terms/pathways with 18,336 available unique genes), KEGG (333 terms/pathways with 8,093 available unique genes), Reactome pathways (2,474 terms/pathways with 10,855 available unique genes), Reactome reactions (13,015 terms/pathways with 10,855 available unique genes), and Wiki Pathways (667 terms/pathways with 7,633 available unique genes).

### RNA Sequencing (RNA-Seq) Data and Statistical Analysis

As we have reported previously, human aortic endothelial cells (HAECs) were treated with vehicle control or lysophosphatidylinositol (LPIs, 16:0) (10 μM) for 18 h. The RNA-Seq data are available in the Array Express database under accession number *E-MTAB-6605* (19).

The expression changes were listed in the results with *p* < 0.05 (statistical significance). Genes with expression changes more than log2 (1) in our RNA-Seq data were defined as upregulated, while those with expression decrease of more than log2 (1) were defined as downregulated (Supplementary Tables).

## Results

### GPR55, a Specific Receptor for LPIs, Is Expressed on the Endothelium of Both Human and Mouse Aortas and Is Significantly Upregulated in Hyperlipidemia

To significantly improve our understanding of LPI-induced activation of HAECs with focus on EC activation key features, such as membrane protein adhesion and signaling and secretory function, an endothelial biology knowledge (3–5, 23, 24, 33, 34, 57–59)-based transcriptomic profile strategy was formulated, and 11 gene lists with 16,114 genes: (i) a comprehensive list of 373 cluster of differentiation (CD) markers (plasma membrane proteins) identified by specific monoclonal antibodies (https://en.wikipedia.org/wiki/List_of_human_clusters_of_differentiation); (ii) 159 updated EC-specific biomarkers (60); six types of secretomes namely, (iii) canonical secretome with 2,640 genes (encoded by all human genome-encoded proteins with a signal peptide) as we have reported (13); (iv*)* DAMP-sensor caspase-1 (1, 26, 33, 34, 47, 61–65)-gasdermin D (GSDMD) (66) secretome (proteins secreted extracellularly *via* activated caspase-1 cleaved N-terminal GSDMD protein pore) with 961 genes (48, 67); (v) caspase-4-GSDMD secretome (proteins secreted extracellularly *via* activated caspase-4 cleaved N-terminal GSDMD protein pore) with 1,223 genes (48, 68), (vi) exosome secretome with 6,560 genes, as we have reported (48); (vii) a complete list of 1,176 Human Protein Atlas (HPA)-classified cytokines; viii) a complete list of 200 HPA-classified chemokines, as we have reported (49); (ix) a complete list of 165 reactive oxygen species (ROS) regulators (regulatome), as we have reported (50); (x) a complete list of 1,496 human genome-encoded TFs from the Human Protein Atlas, as we have reported (3, 49); finally, (xi) a complete list of 1,158 human nuclear genome DNA-encoded mitochondria genes from the Broad Institute at MIT, were analyzed in this study (Figure 1A). As outlined in Figure 1B, all the examinations on EC membrane proteins, such as EC-specific biomarkers, CD markers, and the six types of secretomes were phenotypic studies. The three molecular mechanisms, namely TFs, mitoCarta genes, and ROS regulatome, were mechanistic studies.

**Figure 2:**
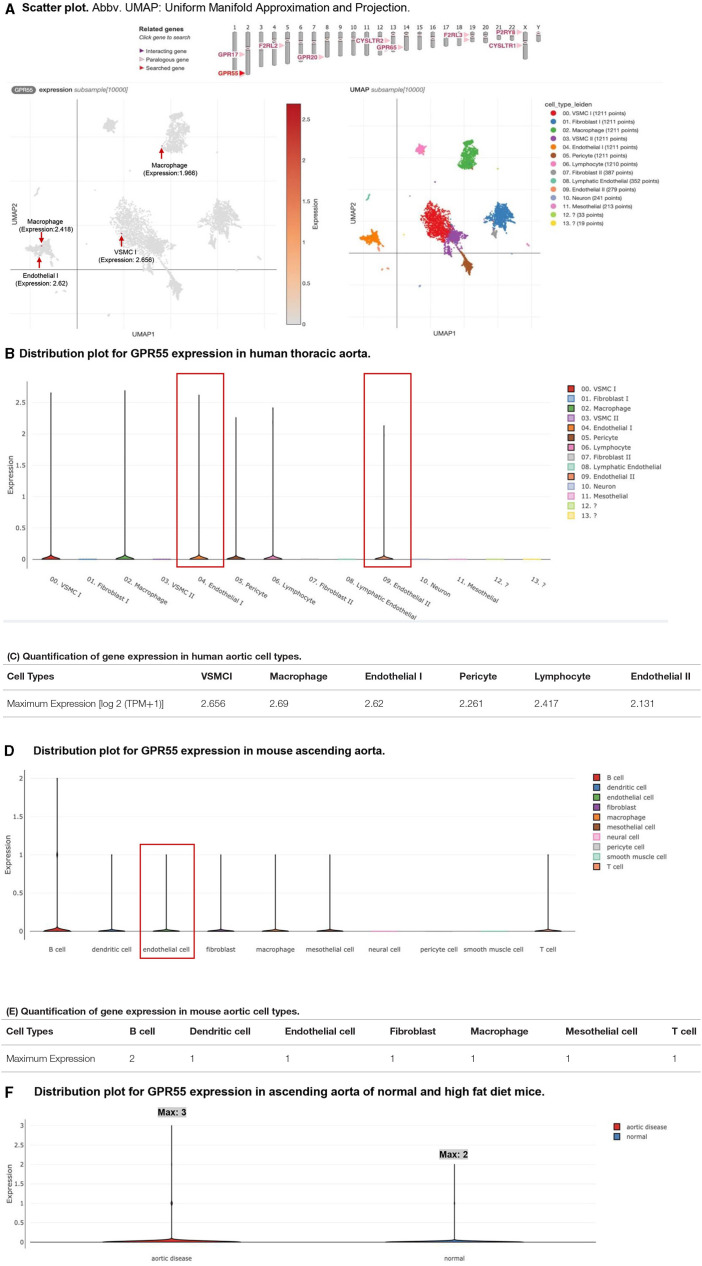
G protein-coupled receptor 55 (GPR55) mRNA transcripts are found in endothelial cells in both human and mouse aortas. Moreover, the expression of GPR55 is upregulated in aortic disease. **(A)** Single-cell RNA-seq analyses were performed on the Single Cell RNA-Seq database of the Broad Institute of MIT and Harvard (Single-Cell RNA-Seq database portal for human thoracic aorta: https://singlecell.broadinstitute.org/single_cell/study/SCP1265/deep-learning-enables-genetic-analysis-of-the-human-thoracic-aorta?genes=GPR55#study-visualize (https://doi.org/10.1101/2020.05.12.091934); GPR55 expressions in macrophage, vascular smooth muscle cells, and endothelial cells of the human aorta are labeled with red arrows in the Scatter. **(B)** GPR55 expressions in endothelial cells of the human aorta were boxed in red in the Distribution. **(C)** The table shows the maximum expressions of GPR55 in each cell type. The expression unit should be the log_2_ (TPM+1). Note: **(A–C)** show the expression of GPR55 in humans. The single-cell transcriptional profiles in **(B,C)** from 54092 thoracic aortic cells singled out 14 clusters representing 13 cell types. **(D)** Single Cell RNA-Seq database portal for mouse study: https://singlecell.broadinstitute.org/single_cell/study/SCP1361/single-cell-transcriptome-analysis-reveals-cellular-heterogeneity-in-the-ascending-aorta-of-normal-and-high-fat-diet-mice?genes=Gpr55#study-visualize). GPR55 expressions in endothelial cells of mouse aorta were boxed in red in the Distribution. **(E)** The table shows the maximum expression of GPR55 in each cell type. **(F)** The expression of GPR55 increased in the high fat-fed aortic disease mouse model compared with the normal control mice. The expression unit should be the log_2_ (TPM+1). Note: The transcriptional profiles in **(D–F)** represent 24,001 aortic cells and 10 cell types.

As we mentioned in the introduction, G protein-coupled receptor 55 (GPR55, 319 amino acids, NIH-NCBI Protein database ID: NP_005674.2) is the specific receptor for LPIs (51, 69). The tissue RNA-Seq data from NIH-NCBI Gene database ID 9290 (https://www.ncbi.nlm.nih.gov/gene/9290) showed that significant GPR55 expressions (>0.5 reads per kilobase million, RPKM) were found in six tissues, such as the appendix, duodenum, lymph node, small intestine, spleen, and testis among 27 human tissues from 95 human individuals (Supplementary Figure 1). The expression of GPR55 was found in the human heart, although the GPR55 expression data from the vessel were not listed. However, the expressions of GPR55 in human and mouse aortic endothelial cells remained unknown. Hence, we hypothesized that GPR55 is expressed in human and mouse aortic endothelial cells. To examine this hypothesis, the human thoracic aorta single-cell RNA-Seq data were analyzed on the Single Cell^Beta^ Portal database of the Broad Institute at Massachusetts Institute of Technology (MIT) and Harvard (https://singlecell.broadinstitute.org/single_cell/study/SCP1265/deep-learning-enables-genetic-analysis-of-the-human-thoracic-aorta?genes=GPR55#study-summary). As shown in Figures 2A,B, the expressions of GPR55 were distributed in six aortic cell clusters identified in 54,092 cells, such as vascular smooth muscle cells, fibroblasts, macrophages, endothelial cells, pericytes, and lymphocytes. Of note, GPR55 expression in both subsets of EC made EC the only cell type with GPR55 expression among all subsets of the cell type (Figure 2B). The maximum GPR55 expression in EC reached 2.62 log_2_ (transcripts per million, TPM+1), ranking third among all the six cell types (Figure 2C). In addition, GPR55 was also expressed in ECs of the mouse aorta. Transcriptions of 24,001 aortic cells were profiled, and ten aortic cell types were identified (https://singlecell.broadinstitute.org/single_cell/study/SCP1361/single-cell-transcriptome-analysis-reveals-cellular-heterogeneity-in-the-ascending-aorta-of-normal-and-high-fat-diet-mice?genes=Gpr55#study-summary). GPR55 mRNA transcripts were found in B cells, dendritic cells, endothelial cells, fibroblasts, macrophages, mesothelial cells, and T cells of mouse aortas (Figures 2D,E). However, no significant expression of GPR55 was found in aortic neural cells, pericyte cells, and smooth muscle cells (Figure 2D). Moreover, GPR55 mRNA transcripts in aortic cells were expressed at much higher levels in the aortas of high-fat-fed mice than in the aortas of normal chow diet-fed healthy control mice (Figure 2F).

Taken together, these findings have demonstrated that first, LPI receptor GPR55 is expressed in human and mouse aortic endothelial cells; second, GPR55 is also expressed in human aortic vascular smooth muscle cells, fibroblasts, macrophages, pericytes, and lymphocytes, and mouse aortic B cells, dendritic cells, fibroblasts, macrophages, mesothelial cells, and T cells. Of note, the expression patterns of GPR55 in aortic endothelial cells, fibroblasts, macrophages, and lymphocytes are shared between human aortas and mouse aortas; *third*, high fat diet-induced hyperlipidemia upregulates aortic GPR55 expression, suggesting critical roles of GPR55 in hyperlipidemia-accelerated atherosclerosis (11, 14, 15, 33, 44, 47, 57, 70, 71). The results were well correlated with our report on LPI-induced activation of EC (19).

### LPIs Upregulate 43 Out of 373 Clusters of Differentiation (CD) Markers in HAECs, Promoting EC Activation, Innate Immune Trans-Differentiation, and Immune and Inflammatory Responses; 72.1% of LPI-Upregulated CD Markers Are Not Induced in Three Types of Virus-Infected Human Endothelial Cells

**Figure 3:**
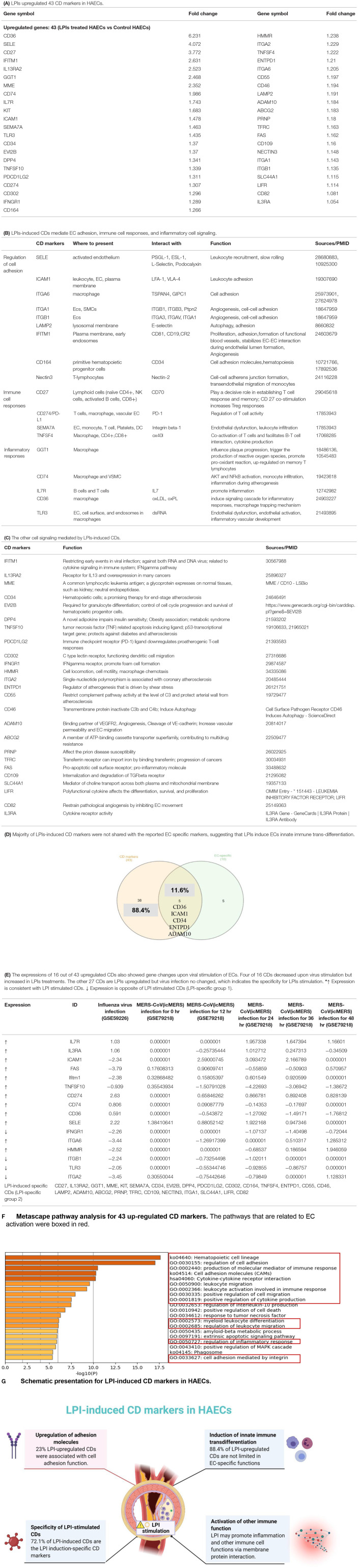
LPIs upregulated EC adhesion molecules and cluster of differentiation (CD) marker-mediated signaling pathways. **(A)** 373 CD markers were used for database mining. Genes with *p* < 0.05 and log_2_ FC > |1| were selected as significantly changed genes. The total number of significantly changed CD markers is 65; upregulated genes account for 66.2% (43/65) and downregulated genes occupied 33.8% (22/65). Downregulated CDs can be found in Supplementary Table S1. **(B)** Eighteen out of 43 LPI-induced CDs mediate EC adhesion, immune cell responses, and inflammatory cell signaling. **(C)** Twenty-five out of 43 LPI-induced CDs mediates the other cell signaling. **(D)** One hundred fifty-nine endothelial cell-specific markers were generated (modified from PMID: 29333215). The LPI-upregulated EC-specific genes were compared with the upregulated CD markers. The Venn diagram indicated that five adhesion molecules showed in the overlapped area between endothelial cell-specific cell markers and LPI-stimulated CD markers. The functions of five overlapped CD markers and most of them participate in the cell adhesion process. **(E)** About 43 upregulated CDs were screened in seven virus-stimulated EC datasets (PMID:34248940). Sixteen out of 43 showed different changes in these seven datasets. **(F)** Upregulated genes of LPI-treated HAECs were analyzed by Metascape (https://metascape.org/gp/index.html#/main/step1). Pathways with high expression are related to the cell adhesion process, leukocyte migration, and inflammation. **(G)** A schematic presentation shows how LPIs regulate endothelial cells by mediating membrane protein interactions. *(G) was created with Biorender.com.

Our recent report showed that LPIs upregulate the expressions of membrane proteins, such as E selectin (SELE), intercellular adhesion molecule 1 (ICAM1), CD74, human leukocyte antigen (HLA) allele-DRB1, and HLA-DMA in HAECs (19). EC expresses specific clusters of differentiation (CDs), such as CD31, which includes various membrane-bound or cytoplasmic molecules on its surface, helps in easier identification of ECs from other cell types, such as CD4^+^ T cells (72–77), and can be defined by specific monoclonal antibodies (78). However, the overall LPI-modulated membrane protein expressions remained unknown. An excellent review summarized that 11 CDs expressed in ECs, namely, CD54 (ICAM1), CD102 (ICAM2), CD146 (MCAM), CD322 (JAM-B), CD106 (VCAM1), CD31 (PECAM1), CD155 (poliovirus receptor), CD99 (MIC2), CD62E (E-selectin), CD62P (P-selectin), and CD144 (VE-Cadherin), are involved in monocyte trafficking across the vessel wall (79). However, an important question remained whether the expression of all the other CDs is modulated in EC activation. We hypothesize that LPIs play a vital role in modulating the expressions of CDs and other EC adhesion molecules. To examine this hypothesis and study how LPIs change immunophenotyping and alter the behavior of ECs, we collected a complete list of 373 CD markers from a human protein database (https://www.proteinatlas.org/search/protein_class:CD+markers) and screened these CD markers in our LPI-treated HAEC RNA-Seq dataset (19). By comparing the RNA-Seq data of the LPI-treated HAECs with that of untreated HAEC controls, 21,252 genes were found to be significantly modulated (*p* < 0.05, |log 2FC| ≥ 1). As shown in Supplementary Table S1, 65 out of 373 (17.4%) CDs showed significant expression changes in LPI-treated HAECs. Among them, 43 CDs out of 373 (11.5%) were dramatically upregulated (Figure 3A). Of the 43 upregulated CD markers, we found that nine were involved in the regulation of cell adhesion, namely, selectin E (SELE, CD62E), intercellular adhesion molecule-1 (ICAM1, CD54, which are ligands for the leukocyte adhesion protein LFA-1), integrin a6 (ITGA6, CD49f, and beta4, which promote tumorigenesis where beta1 inhibits erbB2/HER2 signaling), ITGA1 (CD49a, which is involved in cell adhesion, inflammation, and fibrosis), ITGB1 (CD29, which is involved in cell adhesion and recognition in various processes such as embryogenesis, hemostasis, tissue repair, immune response, and cancer metastasis), lysosome-associated membrane protein 2 (LAMP2 and CD107b, which play an important role in chaperone-mediated autophagy), interferon-induced transmembrane protein 1 (IFITM1, which inhibits the entry of viruses, viral fusion, and release to the cytoplasm), CD164 (multi-glycosylated core protein 24, which regulates the proliferation, adhesion, and migration of hematopoietic progenitor cells), and nectin cell adhesion molecule 3 (nectin3 and CD113, which function as adhesion molecules at adherens junctions). These CDs were also functional in leukocyte recruitment, cell-cell interaction, and slow rolling (80–90). The second group of five upregulated CDs, namely CD27 (a tumor necrosis factor receptor (TNFR), a superfamily member and co-stimulation receptor), semaphoring 7A (SEMA7A), CD108, (which promotes axonal growth and T cell development), TNFSF4 (CD134, OX40 ligand, a co-stimulation receptor), and GGT1[CD224, which promotes clear cell renal cell carcinoma initiation and progression (91)], played roles in co-stimulating T cell immune responses, promoting cancer growth (92), and establishing immune memory (93–97). In addition, the third group of four inflammation-related CDs, such as MHC HLA-DR gamma chain (CD74) for MHC class II antigen presentation, interleukin-7 receptor (IL7R, which plays a critical role in the development of lymphocytes in a process called VDJ recombination), scavenger receptor class B, member 3 (CD36) for oxidized low-density lipoprotein (oxLDL) cell internalization (98), and toll-like receptor 3 (TLR3) for binding to double-stranded RNA/unmethylated CpG DNA and cooperating with scavenger receptor SREC-I to trigger inflammatory innate immune response (99), were dramatically upregulated in LPI-treated HAECs (Figure 3B) (100–103). Moreover, the fourth group included 25 CDs involved in many cellular functions, such as viral infection (IFITM1), interferon-gamma receptor signaling (IFITM1, IFNGR1), growth factor/cytokine receptors (TNFSF10, ADAM10, TFRC, FAS, CD109, IL13RA2, IL3RA, LIFR, DPP4), immune checkpoint receptors (PDCD1LG2), complement signaling (CD55, CD46), and hematopoiesis and stem cells [CD34, EVI2B, and KIT (104)] (Figure 3C).

To better understand the alteration of endothelial cell surface markers induced by LPIs, we further gathered a list of 159 endothelial cell-specific biomarkers (60) (Supplementary Table S2). Figure 3D shows that five CD markers (11.6%) out of 43 LPI-upregulated CDs were shared with 5 out of 10 LPI-upregulated EC-specific cell biomarkers: CD36, ICAM1, CD34, ectonucleoside triphosphate diphosphohydrolase 1 (ENTPD1), and ADAM metallopeptidase domain 10 (ADAM10). Among these five CD markers, ICAM1, CD34, and ADAM10 directly mediate cell-cell adhesion. For example, the classic adhesion molecule ICAM1 on the surface of EC could interact with the molecule LFA-1 on lymphocytes, leading to a pro-inflammatory signaling cascade (82). CD34, a marker for human hematopoietic progenitor cells, exhibits E-selectin binding activity, facilitating leukocyte rolling and adhesion (105). ADAM10 showed a significantly high expression in atherosclerotic plaque, and its activity was necessary for chemotaxis/migration of monocytes and ECs (106). Of note, the LPIs upregulating 38 out of 43 CDs (88.4%) that did not overlap with EC-specific markers suggested that as high as 88.4% of the CDs upregulated by LPIs are functional in various aspects and not limited to EC-specific functions. These results have demonstrated that the LPI stimulation of aortic ECs may induce innate immune trans-differentiation of ECs, as we have reported (19), and non-EC-specific functions.

In order to identify CD markers specifically induced by LPIs, we examined the expressions of 43 LPI-induced CD markers in the microarray datasets of seven virus-infected human endothelial cells, such as influenza virus-infected human umbilical vein endothelial cells (HUVEC), middle east respiratory syndrome coronavirus (MERS-CoV, homologous to severe acute respiratory syndrome coronavirus 2, SARS-CoV2, or COVID-19)-infected human microvascular endothelial cells, and Kaposi's sarcoma-associated herpes virus(KSHV)-infected human dermal endothelial cells, as we have reported (3). As shown in Figure 3E, the 43 LPI-induced CD markers can be classified into three groups: (1) 12 LPIs were upregulated, and pathogen-associated molecular pattern (PAMP)-triggered (virus-infection) was upregulated (activated endothelial cell shared), namely, IL7R, IL3RA, ICAM1, FAS, Ifitm1, TNFSF10, CD274, CD74, CD36, SELE, ITGA6, and HMMR; (2) 4 LPIs were upregulated, but virus infection was downregulated (LPI-specific group 1), such as IFNGR1, ITGB1, TLR3, and ITGA2; 3) 27 LPIs were upregulated but virus infection was not changed (LPI-specific group 2) namely, CD27, IL13RA2, GGT1, MME, KIT (CD117, stem cell growth factor receptor), SEMA7A (CD108), CD34 (107), EVI2B, DPP4 (CD26, its inhibitors approved for treating type 2 diabetes), PDCD1LG2, CD302, CD164, TNFSF4 (OX40 ligand, CD252), ENTPD1, CD55, CD46, LAMP2, ADAM10, ABCG2 (CDw338, breast cancer resistant protein), PRNP, TFRC, CD109, NECTIN3, ITGA1, SLC44A1, LIFR, and CD82. Of note, future work is needed to determine whether LPIs upregulated CDs share with the CDs upregulated in responses to the stimulation of PAMPs/DAMPs and conditional DAMPs (20, 21).

In addition to analyzing the functions of upregulated CD markers, Metascape was used for pathway analysis (https://metascape.org/gp/index.html#/main/step1) for small gene sets in comparison with that analyzed by IPA. Twenty pathways, using upregulated CD markers from LPI-treated HAECs (Figure 3F), were identified, such as the top 10 functions of hematopoietic cell lineage, regulation of cell adhesion, production of molecular mediator of the immune response, cell adhesion molecules (CAMs), cytokine-cytokine receptor interaction, leukocyte migration, leukocyte activation involved in immune response, positive regulation of cell migration, positive regulation of cytokine production, and regulation of IL-10 production. Among these 20 pathways, 11 were related to EC activation (boxed), namely, the top 2–9 functions mentioned above, and myeloid leukocyte differentiation, regulation of inflammatory response, and cell adhesion mediated by integrin. Of note, the “regulation of cell adhesion” showed extraordinarily high enrichment up to around log10 (16).

Our results on LPI-induced CD markers in HAECs demonstrated that, first, of the total 65 LPI-changed CD markers, 66.2% were significantly upregulated by LPI stimulation, and that only 33.8% were downregulated by LPIs. Among the LPI-upregulated CD markers, 23% were associated with cell adhesion; 9.3 and 9.3% were related to immune response and inflammation, respectively. These results suggest that LPIs induce aortic EC activation through the upregulation of various adhesion molecules, *via* which LPIs may initiate inflammation by recruiting immune cell accumulation; second, by comparing LPI-upregulated CD markers with EC-specific markers, we found that LPI stimulation upregulates CDs that are significantly different from EC-specific markers, suggesting that LPIs may induce the innate immune trans-differentiation of ECs, as we have reported (19), LPI-activated HAECs may carry out many non-EC-specific functions; third, 31 out of the 43 LPI-induced CD markers (72.1%) are LPI induction-specific CD markers that are not induced in three types of virus-infected endothelial cells, which significantly enhance our understanding of CD markers upregulated in activated ECs; fourth, in addition to inducing EC activation, LPI-induced CD markers may promote other immune cell functions and inflammatory responses *via* membrane protein interactions (Figure 3G).

### LPI-Activated Aortic ECs Upregulate Six Types of Secretomic Genes, Canonical Secretome, Caspase-1-Gasdermin D (GSDMD) Non-Canonical Secretome, Caspase-4/11-GSDMD Non-Canonical Secretome, Exosome Non-Canonical Secretome, HPA-Classified Cytokines, and HPA-Classified Chemokines, Which Makes HAECs a Large Secretory Organ for Inflammation, Immune Responses, and Other Functions

**Figure 4:**
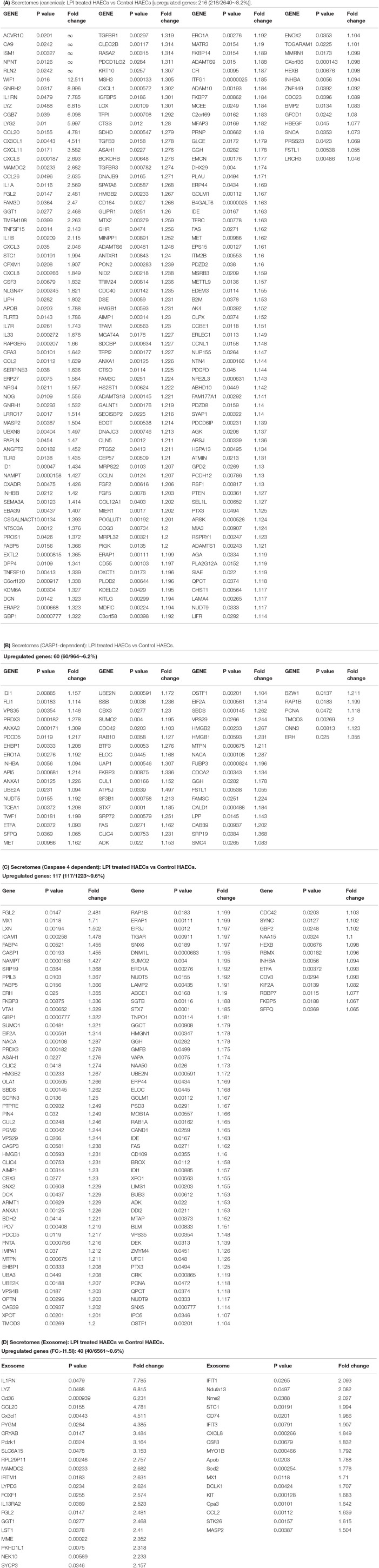
LPIs significantly upregulate secretomic genes of canonical secretomes, non-canonical caspase-1-Gasdermin D (GSDMD), caspase-4-GSDMD, and exosome secretomes in HAECs. **(A)** Among 2,640 canonical secretomes, 216 were significantly upregulated in LPI-treated HAECs. The upregulated genes accounted for 8.2% of the total canonical secretomes. **(B)** Sixty non-canonical caspase 1 dependent secretomes were significantly elevated in LPI-treated HAECs. **(C)** One hundred seventeen non-canonical caspase 4 dependent secretomes were dramatically increased in LPI-treated HAECs. **(D)** Forty exosomes were dramatically increased in LPI-stimulated HAECs. *Genes in **(A–C)** were selected with *p* < 0.05 and log_2_ FC > I1I as significantly changed genes, while genes in exosome listed *p* < 0.05 and log_2_ FC > |1.5| as significantly changed genes. The full list of 923 LPI-upregulated exosomes can be found in Supplementary Table S3. *Created with Biorender.com.

Secretome refers to a collection of actively secreted proteins for a destination outside the nucleus and cytoplasm of the cells. Those proteins are actively transported within the secretory pathways and participate in various signaling functions, such as cytokines, chemokines, adhesion molecules (108), angiogenesis, and wound healing (109). ECs are secretory cells, and protein secretion plays a pivotal role in EC functions, as we and others have reported/reviewed (1, 3, 4, 40, 44, 50, 110, 111). Especially during EC activation, secreted proteins are responsible for cell-cell interaction, affecting vascular tone, cell adhesion, and inflammation (112). The 18 cytokines secreted from EC (110) included pro-inflammatory cytokines, such as tumor necrosis factor-α (TNF-α), interleukin-1 (IL-1), IL-3, IL-5, IL-6, IL-8, IL-11, IL-15, monocyte chemoattractant protein-1 (MCP-1), granulocyte-macrophage colony-stimulating factor (GM-CSF) (3, 57), CD40/CD40L, endothelin-1, regulated upon activation, normal T cell expressed and presumably secreted (RANTES, C-C motif ligand 5, CCL5) and anti-inflammatory cytokine IL1 receptor antagonist (IL1ra), IL10 (59), IL13, transforming growth factor-β (TGF-β), and IL-35 (40, 44, 58, 59, 111). We hypothesize that LPI treatment drives HAEC activation *via* the upregulation of inflammatory and adhesion-related secreted proteins. To gain a comprehensive understanding of how the LPI stimulation of HAECs regulates the secretory functions of ECs, we collected six secretomic gene lists:(1) 2,640 conventional secretomes (with signal peptide) were downloaded from the comprehensive protein database Human Protein Atlas (https://www.proteinatlas.org/), as we have reported (13); (2) 964 non-canonical caspase-1-gasdermin D (GSDMD) secretomes (67); (3) 1,223 non-canonical caspase-4 (humans)/11 (mice) secretomes (68), and 4) 6,560 exosome secretomes downloaded from a comprehensive exosome database (http://www.exocarta.org/) (113), as we have reported (48). As shown in Figure 4A, 216 (8.2%) out of the 2,640 canonical secretomic genes were dramatically increased in LPI-treated HAECs. Similarly, 60 (6.2%) out of the 964 caspase-1-GSDMD non-canonical secretomic genes and 117 (9.6%) out of the 1,223 caspase-4-GSDMD non-canonical secretomic genes were significantly upregulated in the LPI-activated HAECs, respectively (Figures 4B,C). In addition, 40 out of the 6,560 total exosome secretomic genes showed dramatic elevation, with >log2FC 1.5 (fold change) (Figure 4D).

**Figure 5:**
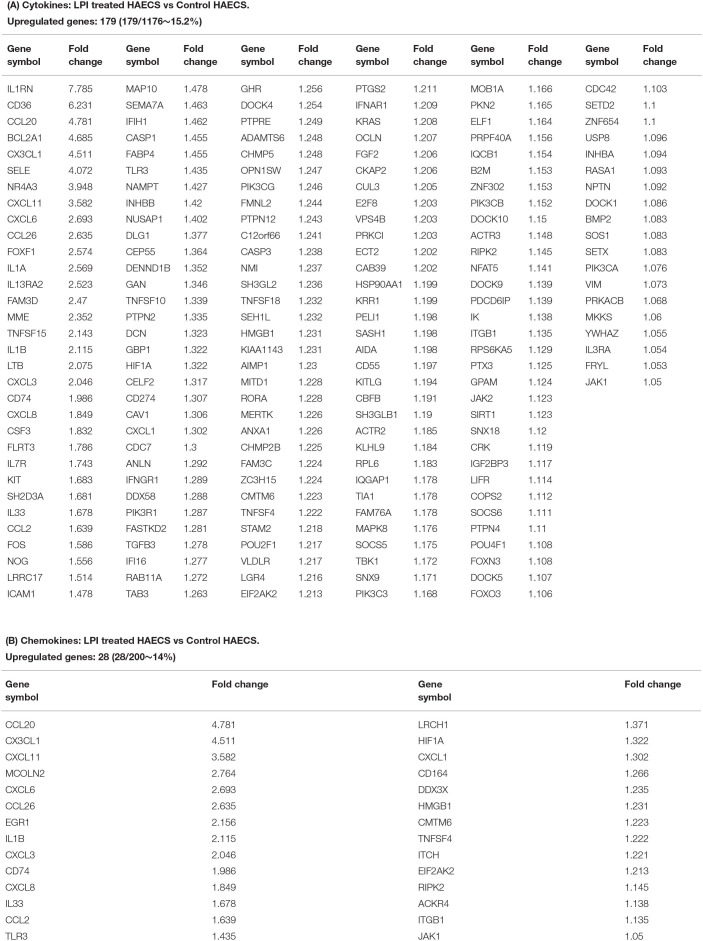
Cytokines and chemokines showed significant upregulation in LPI-treated HAECs compared with the control HAECs. **(A)** One hundred seventy-nine cytokines were significantly upregulated in LPI-treated HAECs. **(B)** Twenty-eight chemokines were significantly upregulated in LPI-treated HAECs.

Of note, secretomes secrete a variety of biologically active molecules, such as (1) growth factors, (2) hormones, (3) cytokines [myokines/exerkines from muscle (113), adipokines from adipose tissues (114), cardiokines from the heart (115), hepatokines from the liver (116), osteokines from bones (116), nephrokines from kidney (113), and neurokines from the brain (113)]; (4) chemokines (117), and (5) many other secretory molecules with poorly characterized functions (13, 48, 49). Cytokines and chemokines released from endothelium have long been documented to be essential in promoting leukocyte recruitment, and inflammation during atherosclerosis, as we and others have reported/reviewed (1, 3, 4, 40, 44, 50, 110, 111). However, the vital question remained whether ECs secrete large pools of cytokines and chemokines during LPI-induced EC activation. Thus, we collected two lists of 1,176 cytokines and 200 chemokines (49) classified by the Human Protein Atlas (HPA, https://www.proteinatlas.org/) and examined the expression changes in the HPA-classified cytokines and chemokines, and their interactors in the LPI-treated HAEC RNA-Seq dataset. Of note, some cytokines and chemokines were overlapped with secretory proteins classified in other secretomes. As shown in Figure 5A, the expressions of 179 (15.2%) out of 1,176 HPA-classified cytokines showed a significant increase, and the expressions of 28 (14%) out of 200 HPA-classified chemokines were significantly upregulated (Figure 5B).

**Figure 6:**
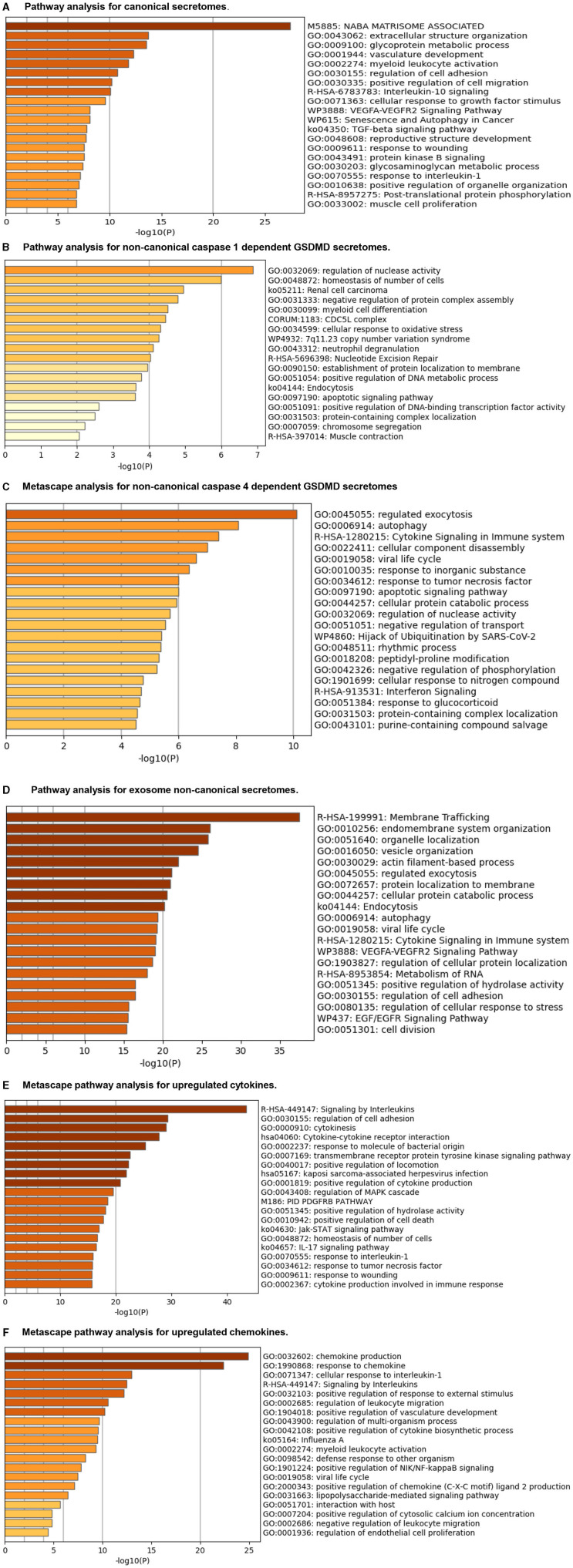
Metascape pathway analysis for upregulated six secretomes and cytokine and chemokines in LPI-treated HAECs. **(A)** Pathway analysis for canonical secretomes. **(B)** Pathway analysis for caspase-1-dependent non-canonical GSDMD secretomes. **(C)** Pathway analysis for caspase-4-dependent non-canonical GSDMD secretomes. **(D)** Pathway analysis for exosomes non-canonical secretomes. **(E)** Metascape pathway analysis for upregulated cytokines. **(F)** Metascape pathway analysis for upregulated chemokines.

The Metascape (https://metascape.org/gp/index.html#/main/step1) database analysis with six types of upregulated secretomes, cytokines, and chemokines in LPI-activated HAECs in Figure 6 demonstrated that the LPI-upregulated canonical secretome had top 10 functional pathways, namely, NABA matrisome associated, extracellular structure organization, glycoprotein metabolic process, vasculature development, myeloid leukocyte activation, regulation of cell adhesion, positive regulation of cell migration, IL-10 signaling, cellular response to growth factor stimulus, and VEGFA-VEGFR2 signaling (Figure 6A). The LPI-upregulated caspase-1-GSDMD non-canonical secretome had top 10 functional pathways, namely, regulation of nuclease activity, homeostasis of the number of cells, renal cell carcinoma, negative regulation of protein complex assembly, myeloid cell differentiation, CDC5L complex, cellular response to oxidative stress, 7q11.23 copy number variation syndrome, neutrophil degranulation, and nucleotide excision repair (Figure 6B). The LPI-upregulated caspase-4-GSDMD non-canonical secretome had top 10 functional pathways, namely, regulated exocytosis, autophagy, cytokine signaling in the immune system, cellular component disassembly, viral life cycle, response to an inorganic substance, response to tumor necrosis factor, apoptotic signaling pathway, cellular protein catabolic process, and regulation of nuclease activity (Figure 6C). The LPI-upregulated exosome non-canonical secretome had top 10 functional pathways, namely, membrane trafficking, endomembrane system organization, organelle localization, vesicle organization, actin filament-based process, regulated exocytosis, protein localization to the membrane, cellular protein catabolic process, endocytosis, and autophagy (Figure 6D). The LPI-upregulated cytokines had top 10 functions, namely, signaling by interleukins, regulation of cell adhesion, cytokinesis, cytokine-cytokine receptor interaction, response to molecule of bacterial origin, transmembrane receptor protein tyrosine kinase signaling, positive regulation of locomotion, Kaposi sarcoma-associated herpesvirus infection, positive regulation of cytokine production, and regulation of MAPK cascade (Figure 6E). The LPI-upregulated chemokines had top 10 functions, namely, chemokine production, response to chemokine, cellular response to interleukin-1, signaling by interleukins, positive regulation of response to external stimulus, regulation of leukocyte migration, positive regulation of vasculature development, regulation of the multi-organism process, positive regulation of cytokine biosynthetic process, and influenza A-related process (Figure 6F).

**Figure 7:**
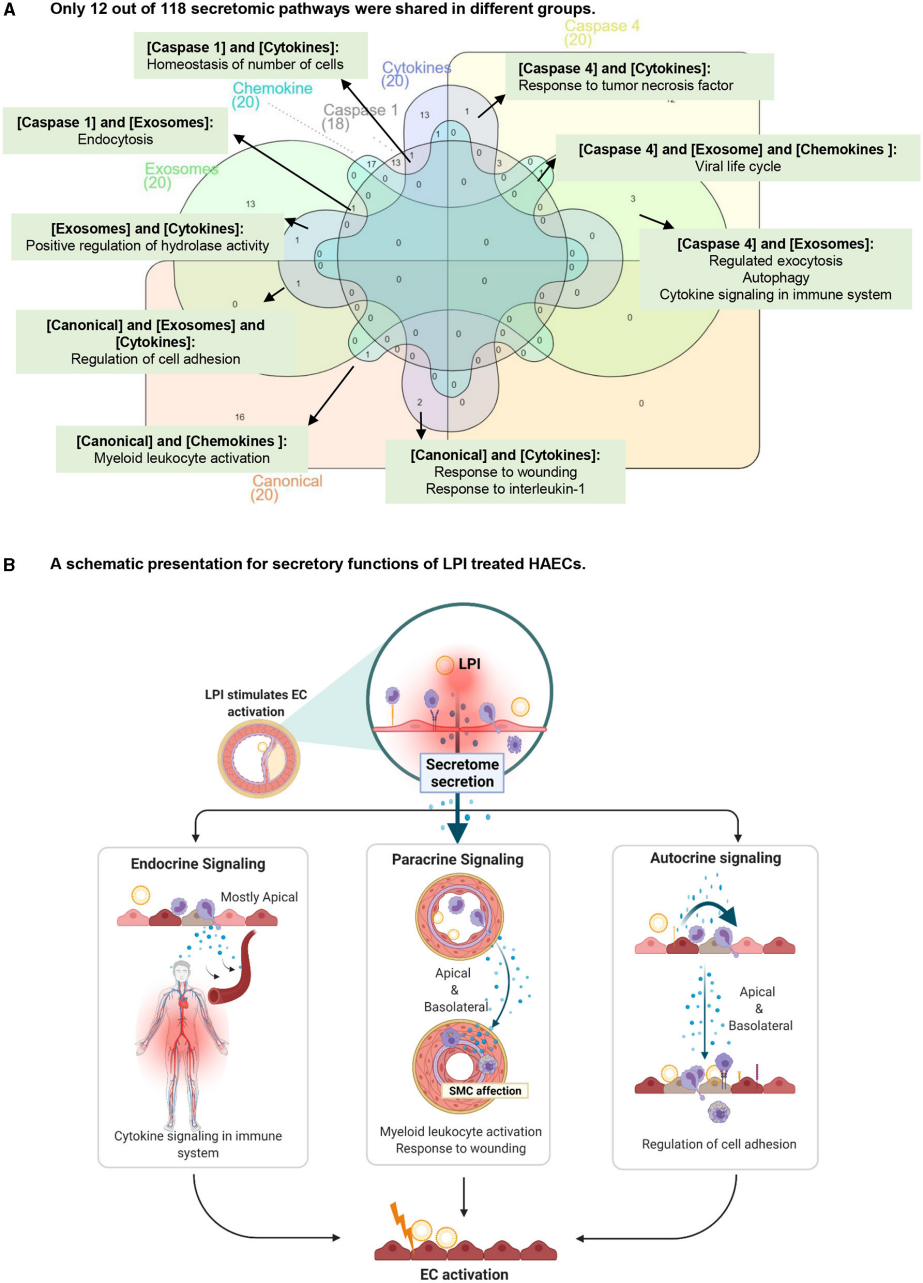
The majority of secretory pathways are mutually exclusive in LPI-stimulated HAECs. **(A)** Venn diagram was used to check the overlapped pathways among six secretomic groups. *(B) was created with Biorender.com.

To find potential connections among the LPI-treated HAEC secretory protein molecules, we created a Venn diagram for the pathways of canonical secretome, caspase-1 secretome, caspase-4 secretome, exosome secretome, HPA cytokines, and HPA chemokines. As shown in Figure 7A, among the 118 secretomic gene pathways identified by the Metascape analysis, the majority of the pathways were secretome-specific; and 12 pathways were shared among the six types of secretomic genes. The caspase-1-GSDMD secretome shared homeostasis of numbers of cells with HPA-cytokines; the caspase-1-GSDMD secretome shared endocytosis with the exosome secretome; the exosome secretome shared positive regulation of hydrolase activity with the HPA cytokines, and three types of secretomes, canonical, exosome, HPA cytokines, shared regulation of cell adhesion; the canonical secretome and HPA chemokines shared myeloid leukocyte activation; the canonical secretome and HPA cytokines shared response to wounding and response to interleukin-1; the caspase-4-GSDMD and exosome secretomes shared three pathways, regulated exocytosis, autophagy, and cytokine signaling in the immune system; three types of secretomes, namely, caspase-4-GSDMD, exosome, and HPA chemokines, shared viral life cycle; caspase-4-GSDMD and HPA cytokines shared response to tumor necrosis factor.

The results have demonstrated for the first time that first, in contrast to the 20 cytokines reported to be secreted from ECs as mentioned above (110), activated aortic ECs are a large secretory organ that can upregulate the transcripts of large numbers (640 genes) of secretory proteins, potentially secrete six long lists of cytokines (179 genes), chemokines (28 genes), and 433 secretomic genes (216 + 60 + 117 + 40 = 433) and modulate the functions of innate and adaptive immune cells, inflammatory cells, other vascular cells, and non-vascular cells *via* three manners, such as autocrine, paracrine, and endocrine (Figure 7B); second, around 10% of secretomes in each category (canonical, caspase-1-GSDMD, caspase-4-GSDMD, exosome, HPA-cytokines, HPA-chemokines) showed significant upregulation after LPI stimulation. The percentages of LPI-upregulated cytokines and chemokines are higher than that of the four types of secretomes, around 15% in each. A similar percentage also occurred in the LPI-stimulated exosome secretomic genes, 923 (923/6,560 in total, ~14.1%) with *p* < 0.05 and log_2_FC >1; third, based on the comparison of top 10 functional pathways related to the LPI-upregulated secretomic genes, canonical secretome, caspase-1-GSDMD non-canonical secretome, caspase-4-GSDMD non-canonical secretome, and exosome non-canonical secretome in LPI-activated aortic EC may carry out different functions in EC adhesion, immune and inflammatory cell activation, regulation of leukocyte migration, regulation of cellular response to stress, and many other functions; fourth, a previous report has suggested that pools of human coronary artery ECs and human umbilical vein ECs have polarized secretomes, such as apical secretome and basolateral secretome. The majority of EC secretomes with 840 proteins and extracellular vesicles (EVs), such as exosome (53)) secretome, are polarized to the apical surface (112). A future proteomic study is needed to determine the polarized secretomes of LPI-activated aortic ECs (Figure 7B).

### LPIs Activate a Transcription Mechanism by Upregulating 172 Transcription Factors, Some of Which, NR4A3, FOS, KLF3, and HIF1A, Play Significant Roles in Promoting Inflammation and Atherosclerosis

**Figure 8:**
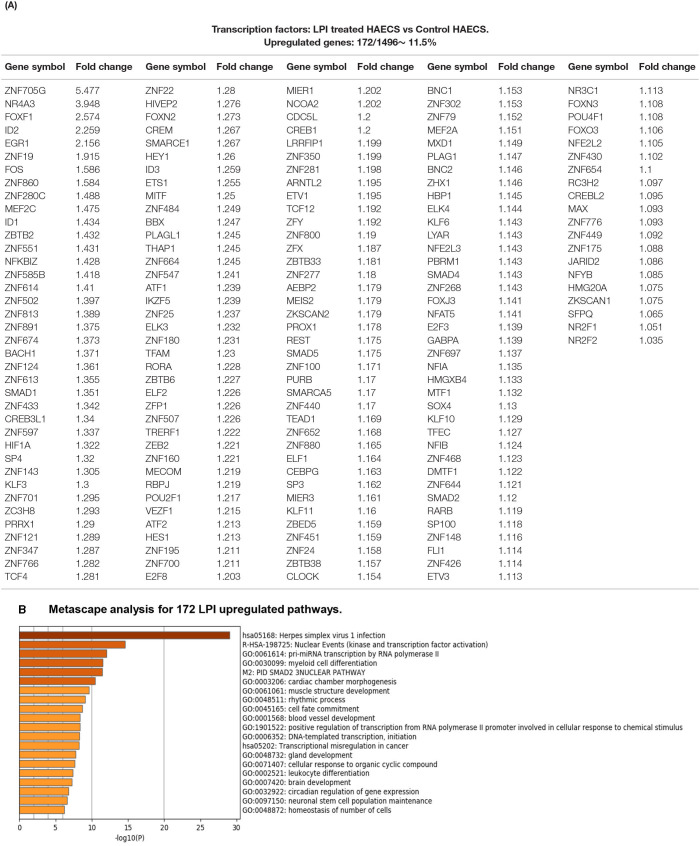
One hundred seventy-two LPI-upregulated transcription factors (TFs) were identified, which mediates 20 pathways after screening for a total of 1,496 TFs. **(A)** Around 11.5% of the genes (172/1496) showed significant upregulation among total TFs. **(B)** Metascape pathways and biological process enrichment analysis for upregulated genes in the LPI group.

To identify molecular mechanisms underlying LPI-induced transcriptomic changes in CDs and EC-specific biomarkers, and six types of secretomic genes, we first examined the LPI-induced transcriptomic remodeling of the master gene transcription factors. We previously reported that three transcription factors (TFs), GATA-binding protein 3 (GATA3), B cell lymphoma 6 (Bcl-6), and histone deacetylase 6 (HDAC6), regulate CD4^+^Foxp3^+^ regulatory T cell (Treg) plasticity and determine Treg conversion into either novel antigen-presenting cell-like Treg or Th1-Treg (118). This result suggests that other T helper cell subsets, such as type 2 CD4+ T helper cell (Th2), and TFs such as GATA3, follicular T helper cell (Tfh) TF Bcl-6, and HDAC6, cooperate with Foxp3 to determine Treg transcriptomes and functions. Moreover, three upregulated TFs, Jun (AP-1 transcription factor subunit), hypoxia-inducible factor-1α (HIF1A), and endothelial PAS domain protein 1 (EPAS1, HIF-2α), collaborate with other pathways and membrane receptors to potentially trans-differentiate CD14^+^ thrombus leukocytes into angiogenic endothelial cells (12). The expressions of 232 transcription regulators are differentially regulated in 28 sets of endothelial cell microarrays in response to the stimulation of a broad spectrum of pathophysiologically relevant pathogen-associated molecular patterns (PAMPs)/danger-associated molecular patterns (DAMPs) (3). We hypothesized that LPIs activate HAECs by upregulating a set of specific TFs. To test this hypothesis, we collected 1,496 TFs from the comprehensive protein database Human Protein Atlas (HPA, https://www.proteinatlas.org/search/cytokine), as we reported recently (13). As shown in Figure 8A, 172 out of the total 1,496 TFs (11.5%, log_2_FC >1, *p* < 0.05) were significantly upregulated in LPI-activated HAECs. In addition, the numbers of LPI-induced upregulation for more than log_2_FC 2 folds, more than log_2_FC 1.5-fold, more than log_2_FC 1.4-fold, more than log_2_FC 1.3-fold, and more than log_2_FC 1.2-fold were 5, 3, 8, 15, and 49 TFs, respectively. Among the highly LPI-upregulated TFs, nuclear receptor subfamily 4 group A member 3 (NR4A3) was a novel target of p53 contributing to apoptosis (119); FoxF1 was a therapy target of Hedgehog-related cancers (120); FOS (AP-1 TF subunit) was one of the TFs linked to ERK/MAPK activation (121), inflammation, and atherosclerosis (122); Kruppel-Like Factor 3 (KLF3) was one of the key mechanosensitive master switches in gene expression in promoting atherosclerosis (123); hypoxia-inducible factor-1α (HIF1A) was a master regulator of EC biology for diabetic atherosclerosis (124).

The Metascape analysis in Figure 8B shows that LPI-upregulated TFs had 20 significant pathways, namely, herpes simplex virus 1 infection, nuclear events (kinase and transcription factor activation), pri-miRNA transcription by RNA polymerase II, myeloid cell differentiation, SMAD2-3 nuclear pathway [main signal transducers for transforming growth factor-β (TGF-β)], cardiac chamber morphogenesis, muscle structure development, rhythmic process, cell fate commitment, blood vessel development, positive regulation of transcription in response to chemical stimulus, DNA-template transcription-initiation, transcription misregulation in cancer, gland development, cellular response to organic cyclic compound, leukocyte differentiation, brain development, circadian regulation of gene expression, neuronal stem cell regulation maintenance, and homeostasis of the number of cells.

Taken together, the results have demonstrated that first, LPIs upregulate 172 (11.5%) out of 1,496 TFs and 80 (5.3%) TFs (log_2_FC > 1.2, *p* < 0.05), suggesting that LPIs have a broad effect on aortic EC transcriptome; second, some LPI-upregulated TFs, such as NR4A3, FOS, KLF3, and HIF1A, play significant roles in promoting inflammation and atherosclerosis; third, other Metascape analysis-identified inflammatory pathways include myeloid cell differentiation, positive regulation of transcription in response to chemical stimulus, cellular response to organic cyclic compound, and leukocyte differentiation.

### LPIs Activate a Mitochondrial Mechanism in Aortic ECs by Upregulating152 Nuclear DNA-Encoded Mitochondrial Genes (MitoCarta) and Promote the Mitochondrial Organization, Respiration, Translation, and Transport

**Figure 9:**
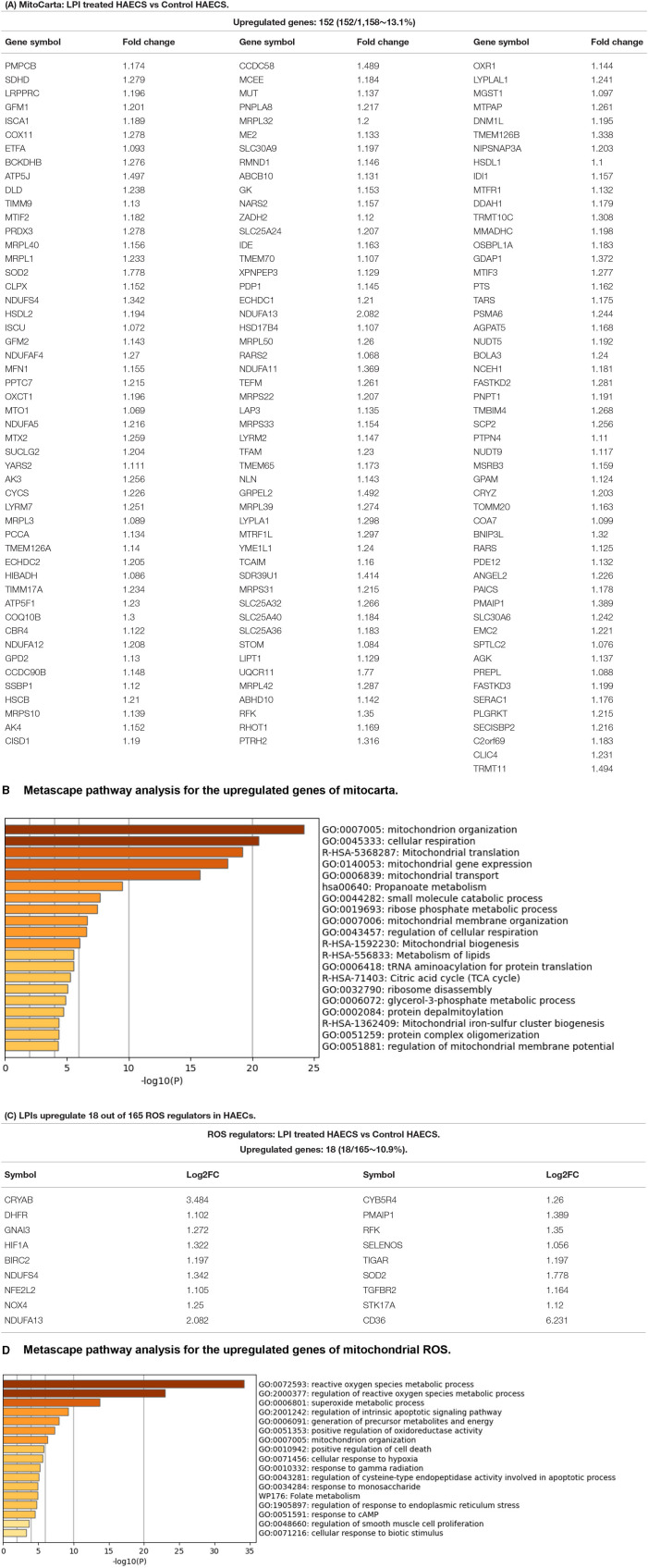
Mitochondrion-related genes showed significant upregulation in LPI-treated HAECs compared with the control HAECs. **(A)** One hundred fifty-two out of 1,158 mitocarta genes were significantly upregulated in LPI-treated HAECs. **(B)** Metascape pathway analysis for the upregulated genes of mitocarta. **(C)** Eighteen mitochondrial reactive oxygen species (ROS) regulators were significantly upregulated in LPI-treated HAECs. **(D)** Metascape pathway analysis for the upregulated genes of mitochondrial ROS.

Our previous reports showed that LPC induces aortic EC activation by increasing mitochondrial reactive oxygen species (mtROS) and proton leaks uncoupled from ATP synthesis (23, 44–46, 125) and that similar to LPC, LPIs also induces the upregulation of ICAM1 and aortic EC activation (19). We hypothesized that LPIs activate aortic ECs *via* a mitochondrion-dependent mechanism and modulate the transcription of genomic (nuclear) DNA-encoded mitochondrial genes (mitoCarta genes). To test this hypothesis, we collected the mitoCarta gene list from the Broad Institute at MIT (https://www.broadinstitute.org/mitocarta/mitocarta30-inventory-mammalian-mitochondrial-proteins-and-pathways). Figure 9A shows that LPIs upregulated 152 (13.1%) out of 1,158 mitoCarta genes. In addition, the Metascape analysis showed that the LPI-upregulated mitoCarta genes had functions of mitochondrion organization, cellular respiration, mitochondrial translation, mitochondrial gene expression, mitochondrial transport, propanoate metabolism, small-molecule catabolic process, ribose phosphate metabolic process, mitochondrial membrane organization, regulation of cellular respiration, mitochondrial biogenesis, metabolism of lipids, tRNA aminoacylation for protein translation, citric acid cycle (TCA cycle), ribosome disassembly, glycerol-3-phosphate metabolic process, protein depalmitoylation, mitochondrial iron-sulfur cluster biogenesis, protein complex oligomerization, and regulation of mitochondrial membrane potential (Figure 9B). Taken together, the results have demonstrated that LPI-activated aortic ECs activate a mitochondrial mechanism by upregulating 152 nuclear DNA-encoded mitochondrial genes (MitoCarta) and promote the mitochondrial organization, cellular respiration, translation, transport, and membrane organization.

### LPIs Activate the Reactive Oxygen Species (ROS) Mechanism in Activated HAECs by Upregulating 18 Out of 165 ROS Regulators

It has been reported that ROS plays a key role in regulating pathophysiological signaling in endothelial cell activation (126) and cardiovascular diseases (127). We also reported that mitochondrial ROS plays a significant role in mediating EC activation (23, 44, 59). In addition, we recently proposed a new working model in which ROS is an integrated cellular network for sensing homeostasis and alarming DAMPs (128). We hypothesized that LPIs modulate the expressions of ROS regulators in HAECs. We collected 165 ROS regulators classified in the Gene Set Enrichment Analysis (GSEA) (https://www.gsea-msigdb.org/gsea/index.jsp) database, as we have reported (50). Figure 9C shows that LPIs upregulated 18 (10.9%) out of 165 ROS regulators in activated HAECs. In addition, the Metascape analysis showed that LPIs upregulated ROS regulators and promoted the functions of ROS metabolic process, regulation of ROS metabolic process, superoxide metabolic process, regulation of intrinsic apoptotic signaling, generation of precursor metabolites and energy, positive regulation of oxidoreductase activity, mitochondrion organization, positive regulation of cell death, cellular response to hypoxia, response to gamma radiation, regulation of cysteine-type endopeptidase activity involved in apoptosis, response to monosaccharide, folate metabolism, regulation of response to endoplasmic reticulum stress, response to cyclic adenosine 3', 5'-monophosphate (cAMP), regulation of smooth muscle cell proliferation, and cellular response to biotic stimulus (Figure 9D). Taken together, the results have demonstrated that first, LPIs upregulate 18 (10.9%) out of 165 ROS regulators in activated HAECs, suggesting that LPIs activate human aortic endothelial cells potentially *via* ROS-mediated mechanisms; second, LPIs upregulate many pathways in regulating ROS metabolic process, mitochondrial metabolism, and cell death.

### Cytoscape Results Have Demonstrated That Three Molecular Mechanisms, Such as 172 LPI-Upregulated TFs, 152 LPI-Upregulated MitoCarta Genes, and 18 LPI-Upregulated ROS Regulators, Are Integrated to Regulate HAEC Activation

**Figure 10:**
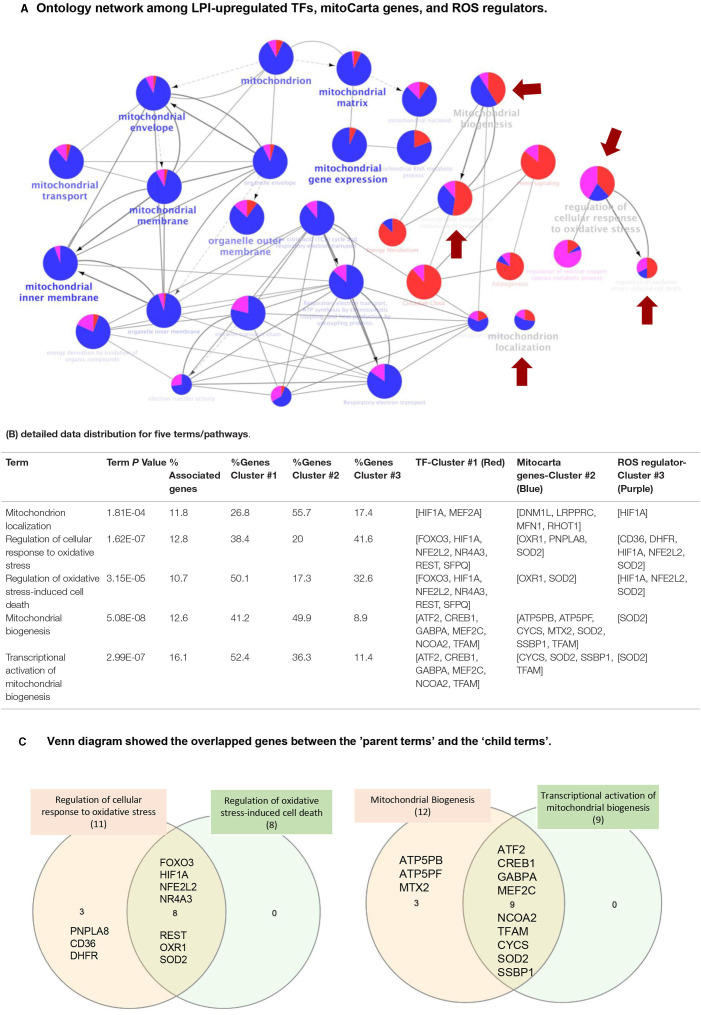
Network data integration database Cytoscape analysis (https://cytoscape.org/) was performed to analyze the network connection among 172 upregulated LPI TFs, LPI-increased mitocarta genes, and LPI-upregulated ROS regulators. **(A)** Ontology network among LPI-upregulated TFs, mitoCarta genes, and ROS regulators. The cluster of 172 LPI-increased TFs is shown in red, the cluster of 152 LPI-upregulated mitoCarta genes is shown in blue, and the cluster of 18 LPI-elevated ROS regulators is shown in purple. One node represents a term/pathway, and the node size stands for significance. The bigger the node size, the higher the significance. (the smallest node in this figure is *p* < 0.05). The proportion of the colors in each node indicates the percentage of genes that contribute to this term/pathway from each cluster. The red arrow highlights five terms/pathways, indicating that the three clusters of genes equally contribute to a term/pathway. We focus on these five pathways, and the other 25 shown in this figure are closely related to the five. The gray lines indicate the connection between each term. The thicker the line, the stronger the correlation. The gray arrow starts from “parent term” to “child term.” The child term is more specialized than its parent term. **(B)** Detailed data distribution for five terms/pathways. The associated genes% represent how many genes from our three clusters matched to the genes of terms in the database. The gene clusters #1, #2, #3 indicate how many percentages of each cluster of genes contribute to the term/pathway. **(C)** Venn diagram shows the overlapped genes between the “parent terms” and the “child terms.”

We further hypothesized that three molecular mechanisms underlying the LPI activation of human aortic endothelial cells can be connected. To examine this hypothesis, we used the Cytoscape (https://cytoscape.org/) database to visualize and integrate the complex network among 172 LPI-upregulated TFs, 152 LPI-upregulated mitoCarta genes, and 18 LPI-upregulated ROS regulators. As shown in Figure 10A, three groups of genes are loaded in the function ClueGO of the Cytoscape database, and the visual style is set as the clusters with assigned colors. The three groups of genes included 172 LPI-upregulated TFs (shown in cluster 1, red), 152 LPI-elevated Mitocarta genes (shown in cluster 2, blue), and 18 LPI-increased ROS regulators (shown in cluster 3, purple). In the search for potential connections between three color clusters, two selection criteria were used. First, the GO tree interval was set between GO levels 4–10 to identify the representative and specific pathways, meaning mapped genes represent 4 to 50% of the total associated genes. When the pathways were selected to be only presented when the *p*-value of the pathway was less than .05, 185 terms/pathways were identified. The second criteria/step were to find potential connections among the lists of LPIs stimulated TF (Red Cluster), MitoCarta genes (Blue Cluster), and ROS regulators (Purple Cluster). Thus, the genes in all three clusters (Red, Blue, Purple colors) were selected for further analysis. After the first and second screening, five terms/pathways were chosen that genes associated with the term/pathways were from different, overlapping clusters (all clusters <60%). The representative genes are shown in Figure 10B, and include: (i) mitochondrial biogenesis (13% associated genes to the term, 41% for cluster 1, 50% for cluster 2, and 9% for cluster3); (ii) regulation of cellular response to oxidative stress (13% associated genes to the term, 38% for cluster 1, 20% for cluster 2, and 42% for cluster3); (iii) regulation of oxidative stress-induced cell death (11% associated genes to the term, 50% for cluster 1, 17% for cluster 2, and 33% for cluster3); (iv) transcriptional activation of mitochondrial biogenesis (16% associated genes to the term, 52% for cluster 1, 36% for cluster 2, and 11% for cluster3); and (v) mitochondrion localization (12% associated genes to the term, 27% for cluster 1, 56% for cluster 2, and 17% for cluster 3). Figure 10C shows the overlapped genes between each term. Taken together, the Cytoscape results have demonstrated that three molecular mechanisms, such as 172 LPI-upregulated TFs, 152 LPI-upregulated mitoCarta genes, and 18 LPI-upregulated ROS regulators, are integrated to promote HAEC activation.

## Discussion

We proposed a novel concept that ECs are innate immune cells. Inflammatory mechanisms and endothelial cell activation play essential roles in promoting the progression of cardiovascular diseases, inflammatory diseases, autoimmune diseases, transplantation immune responses, cancer metastasis, and aging diseases (1, 3–6, 23, 33, 34, 44, 47, 57, 58, 61, 70, 92, 129). Significant progress has been made in elucidating molecular mechanisms underlying endothelial cell activation. However, several important issues remain to be addressed: (1) whether aortic endothelial cell activation induces conditional DAMP (20, 21). LPIs upregulate additional membrane proteins for signaling in addition to mediating inflammatory cell adhesion to EC and trans-EC migration; (2) how many secretory proteins can be upregulated during aortic EC activation, and whether aortic ECs are equipped to upregulate various secretomes during EC activation induced by LPIs; (3) whether LPIs activate aortic ECs *via* remodeling ROS regulatome, mitochondrial reprogramming, and TF machinery reshaping. To address these questions, we developed an EC biology knowledge-based transcriptomic formula to analyze RNA-Seq data in a panoramic manner. We made the following important findings: first, GPR55, a specific receptor for LPIs, is expressed in the endothelium of both human and mouse aortas, and is significantly upregulated in hyperlipidemia; second, LPIs upregulate 43 out of 373 clusters of differentiation (CDs) markers in HAECs, promoting EC activation, innate immune trans-differentiation, and immune and inflammatory responses; and 72.1% of LPI-upregulated CD markers are not induced in three types of virus-infected human endothelial cells; third, LPI-activated aortic ECs upregulate six types of secretomic genes, canonical secretome, caspase-1-gasdermin D (GSDMD) non-canonical secretome, caspase-4/11-GSDMD non-canonical secretome, exosome non-canonical secretome, HPA-classified cytokines, and HPA-classified chemokines, which makes HAECs a large secretory organ for inflammation, immune responses, and other functions; fourth, LPIs activate a transcription mechanism by upregulating 172 TFs, some of which, namely, NR4A3, FOS, KLF3, and HIF1A, play significant roles in promoting inflammation and atherosclerosis; fifth, LPIs activate a mitochondrial mechanism in aortic ECs by upregulating 152 nuclear DNA-encoded mitochondrial genes (MitoCarta) and promote mitochondrial organization, cellular respiration, translation, and transport, and membrane organization; sixth, LPIs activate reactive oxygen species (ROS) mechanism in activated HAECs by upregulating 18 out of 165 ROS regulators; seventh, the Cytoscape analysis results have demonstrated that three novel molecular mechanisms, namely, 172 LPI-upregulated TFs, 152 LPI-upregulated mitoCarta genes, and 18 LPI-upregulated ROS regulators, are integrated to regulate HAEC activation.

Our findings on hyperlipidemia-increased GPR55 expression in mouse aortas were correlated with several reports: (1) patients with Crohn's disease (a type of inflammatory bowel disease) manifest higher (12.5-fold) GPR55 mRNA expression in inflamed compared with non-inflamed colonic tissues (*p* < 0.0001) (130); (2) circulating LPIs and the liver expression of GPR55 are upregulated in patients with nonalcoholic steatohepatitis (NASH); the *in vivo* knockdown of GPR55 is sufficient to improve liver damage in mice fed with a high-fat diet and in mice fed with a methionine-choline-deficient diet (131); and *3)* O-1602, the most potent agonist of GPR55, induces lipid accumulation in hepatocytes, which is reversed by treatment with CID16020046, an antagonist of GPR55 (132). Our findings on the LPI upregulation of 640 secretomic genes in activating HAECs and promoting inflammation were well correlated with several reviews (69) and reports: GPR55 antagonist CID16020046 protects oxLDL-induced inflammation in HAECs (133); LPIs, especially the albumin-bound form, induce pro-inflammatory cytokines TNF-a and IL-6 in macrophages *via* the GPR55/MAPKP38 pathway (134); GPR55 antagonist has anti-inflammatory effects in LPS-activated primary microglial cells (135); GPR55 knockout mice show reduced inflammation scores as compared with wild-type mice in an intestinal inflammation model (2.5% dextran sulfate sodium model) (136). Our findings on the LPI upregulation of 172 transcription factors in activated HAECs were well-correlated with the previous report that LPIs induce the activation of several TFs, such as nuclear factor of activated T-cells (NFAT), nuclear factor κ of activated B cells (NF-κB), and serum response element, translocation of NFAT and NF-κB, and GPR55 internalization (137). Of note, GPR55 is a non-cannabinoid receptor 1 or 2 (CB1/CB2) receptor that exhibits affinity for endogenous plant and synthetic cannabinoids. It was reported that LPI-mediated calcium release and mitogen-activated protein kinase (MAPK)-extracellular signal-regulated kinase (ERK) activation depend on the stable expression of GPR55 and that LPIs cannot have the above-mentioned calcium release and MAPK/ERK activation when CB_1_ or CB_2_ is expressed in the cells (137), suggesting the contexture (cannabinoid receptor 1 or 2 expression levels) dependence of LPI pro-inflammatory functions.

**Figure 11:**
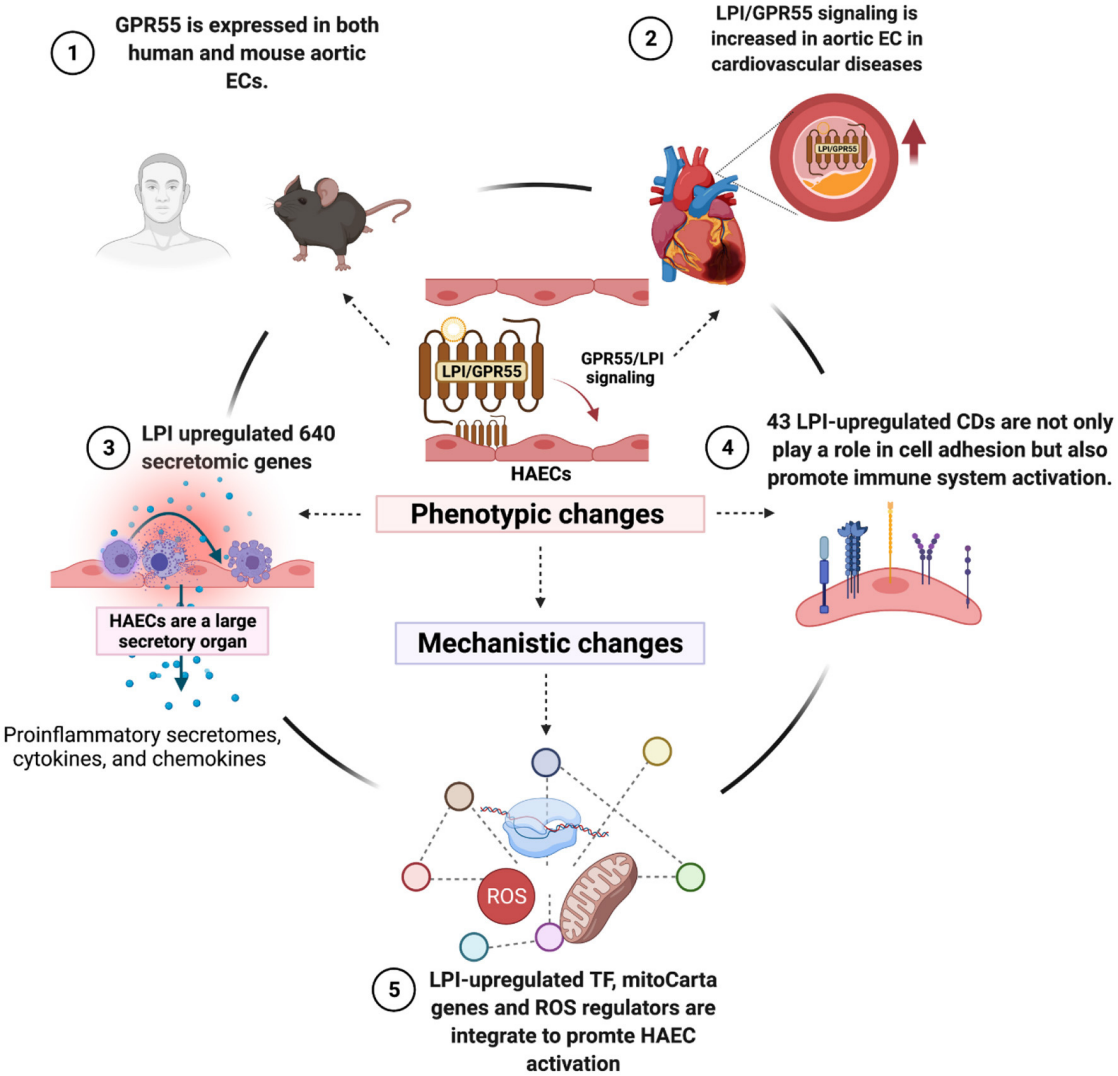
Working model. LPI receptor GPR55 is expressed in both human and mouse aortic endothelial cells. LPIs/GPR55 signaling is upregulated in aortic endothelial cells in hyperlipidemia. We first proposed human aortic endothelial cells as a large secretory organ, which can mediate up to 640 secretomic genes upon LPI stimulation. A large number of secretory proteins contribute a significant role in mediating EC activation and inflammation. LPI-stimulated specific CD markers not only participate in cell adhesion but also play an essential role in immune cell activation, proliferation, and differentiation. All these phenotypic changes may be caused by the mechanisms of synergy among LPI-increased TFs, mitoCarta genes, and ROS regulators. *This figure was created with Biorender.com.

As shown in Figure 11, we proposed a novel working model to integrate all the findings. First, LPI receptor GPR55 is expressed in human and mouse aortic endothelial cells as well as other aortic cell types and is upregulated in hyperlipidemic conditions, suggesting that LPIs/GPR55 signaling is increased in aortic endothelial cells in cardiovascular diseases such as hyperlipidemia. In addition, LPI pro-inflammatory functions may depend on the contexture (cannabinoid receptor 1 or 2 expression levels). Second, by screening 12,763 secretory protein genes in six types of secretomes, we have demonstrated for the first time that human aortic endothelial cells are a large secretory organ. Under stimulation by LPIs, a prototypic conditional DAMP, pro-inflammatory lipid, and human aortic endothelial cells can upregulate as many as 640 secretomic genes *via* six types of secretomic mechanisms, namely, canonical secretome with all human proteins having a signal peptide *via* exocytic direction along the endoplasmic reticulum-Golgi-plasma membrane route, caspase-1-GSDMD non-canonical secretome without a signal peptide but secreted *via* the N-terminal Gasdermin D protein pore/channel, caspase-4(humans)/11 (mice)-GSDMD non-canonical secretome without a signal peptide but secreted *via* the N-terminal Gasdermin D protein pore/channel, exosome non-canonical secretome without a signal peptide but secreted *via* exosomes and docking on target cells with exosome docking mechanism but not cytokine/chemokine receptors, and HPA-classified cytokines and chemokines. In contrast to 18 traditional EC-secreted cytokines and chemokines (110), such as TNF-α, IL-1, IL-3, IL-5, IL-6, IL-8, IL-11, IL-15, MCP-1, GM-CSF (3, 57), CD40/CD40L, endothelin-1, RANTES, IL1ra, IL10 (59), IL13 and TGF-β, and IL-35 (40, 44, 58, 59, 111), these large numbers of secretomic proteins play significant roles in promoting EC activation, inflammatory cell and immune cell recruitment, cancer cell metastasis, immune cell development and regulation, vascular smooth muscle cell function regulation, and many other functions *via* autocrine, paracrine, and endocrine manners, either by apical secretion and/or basolateral secretion. Third, by screening 373 clusters of differentiation markers and 159 EC-specific biomarkers, we have demonstrated for the first time that LPIs upregulate 43 CD markers, five of which are shared with 159 EC-specific biomarkers, and 12 of which are shared with other human endothelial cell activation induced by an influenza virus infection, MERS-CoV infection, and KSHV infection, respectively. In contrast to traditional EC adhesion molecules, such as ICAM1, VCAM1, and SELE, as we and others have reported (33, 58), the 43 LPI-upregulated CD markers not only play significant roles in endothelial cell adhesion and inflammatory and immune cell recruitment but also promote inflammatory cell and immune cell activation, proliferation, differentiation, and immune tolerance. Fourth, three novel molecular mechanisms, namely, 172 LPI-upregulated transcription factors, 152 LPI-upregulated mitoCarta genes, and 18 LPI-upregulated ROS regulators, are integrated to promote HAEC activation.

Our results have provided novel insights into aortic endothelial cell (EC) activation, formulated an EC biology knowledge-based transcriptomic profile strategy, and identified new targets for the future development of therapeutics for cardiovascular diseases, inflammations, immune diseases, transplantation, aging, and cancers. One limitation of all the RNA-Seq data analyses is that due to the low-throughput nature of verification techniques in every laboratory, including ours, we could not verify every result we found with the analyses of high-throughput data, which are similar to all the studies with RNA-Seq (19, 59), single-cell RNA-Seq, metabolomics (23), chromatin immunoprecipitation (CHIP)-Seq (24, 44), and other-omics data (11, 138, 139). We acknowledge that carefully designed *in vitro* and *in vivo* experimental models will be needed in the future to verify the LPI-upregulated genes further and the underlying mechanisms we report here (9, 140).

## Data Availability Statement

The datasets presented in this study can be found in online repositories. The names of the repository and accession numbers can be found below: National Institutes of Health (NIH), National Center for Biotechnology Information (NCBI), Gene Expression Omnibus (GEO) DataSets database (https://www.ncbi.nlm.nih.gov/gds), GSE 59226 (Influenza virus infection), GSE 79218 (MERS-CoV infection for 0, 12, 24, 36, 48 h), and GSE 1377 (Kaposi's Sarcoma associated herpes virus).

## Author Contributions

KX carried out data gathering and data analysis and prepared the tables and figures. YSh, FS, AG, CD, LL, YL, YSu, HX, DP, XQ, JS, EC, XJ, and HW aided in the analysis of data. XY supervised the experimental design, data analysis, and manuscript writing. All the authors read and approved the final manuscript.

## Funding

This study was partially supported by NIH grants to HW and XY.

## Conflict of Interest

The authors declare that the research was conducted in the absence of any commercial or financial relationships that could be construed as a potential conflict of interest.

## Publisher's Note

All claims expressed in this article are solely those of the authors and do not necessarily represent those of their affiliated organizations, or those of the publisher, the editors and the reviewers. Any product that may be evaluated in this article, or claim that may be made by its manufacturer, is not guaranteed or endorsed by the publisher.
