# Supplementary material for: Novel Knowledge-Based Transcriptomic Profiling of Lipid Lysophosphatidylinositol-Induced Endothelial Cell Activation
Source: Front Cardiovasc Med. 2021 Nov 29;8:773473. doi: 10.3389/fcvm.2021.773473 (PMC8668339; doi:10.3389/fcvm.2021.773473)

Supplementary Figure 1. Human RNA-seq normal tissues for GPR55 from NIH-NCBI Gene database ID 9290. Abbv. RPKM: Reads per kilo base per million mapped reads. In order to determine tissue-specificity of all protein-coding genes, RNA-seq was performed of tissue samples from 95 humans. The average expression of GPR55 in heart from 4 individuals was RPKM 0.034 ± 0.017.

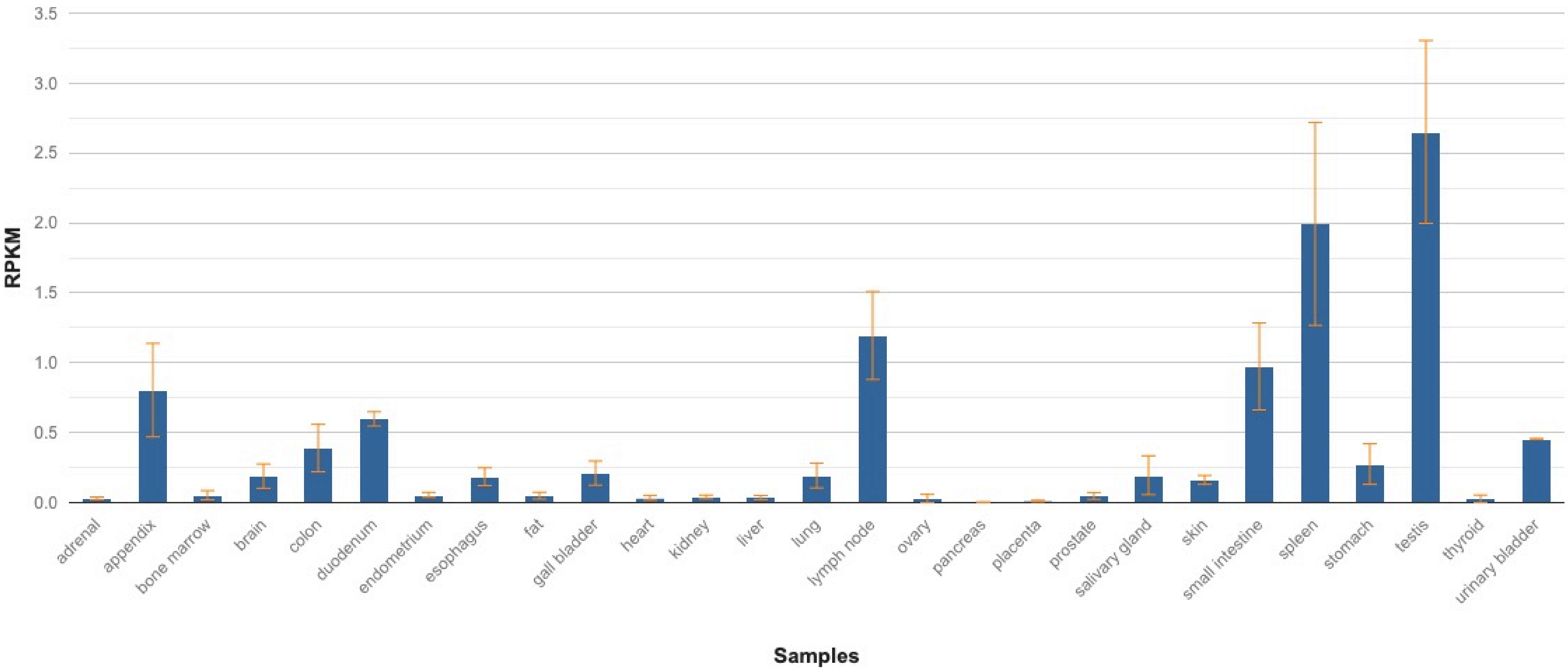

Supplement: Supplementary file 2 [file Image_1.pdf]
